# Probiotics suppress nonalcoholic steatohepatitis and carcinogenesis progression in hepatocyte-specific PTEN knockout mice

**DOI:** 10.1038/s41598-022-20296-3

**Published:** 2022-09-28

**Authors:** Naoshi Arai, Kouichi Miura, Kenichi Aizawa, Mariko Sekiya, Manabu Nagayama, Hirotsugu Sakamoto, Hiroshi Maeda, Naoki Morimoto, Sadahiko Iwamoto, Hironori Yamamoto

**Affiliations:** 1 Department of Medicine, Division of Gastroenterology, Jichi Medical University, 3311-1 Yakushiji Shimotsuke, Tochigi, 329-0498 Japan; 2 Division of Clinical Pharmacology, Department of Pharmacology, Jichi Medical University, 3311-1 Yakushiji Shimotsuke, Tochigi, 329-0498 Japan; 3 Division of Human Genetics, Center for Community Medicine, Jichi Medical University, 3311-1 Yakushiji Shimotsuke, Tochigi, 329-0498 Japan

**Keywords:** Microbiology, Gastroenterology

## Abstract

Nonalcoholic fatty liver disease (NAFLD), a hepatic characteristic of metabolic syndrome, received significant attention in clinical settings. The multiple-hit theory is one of the proposed mechanisms of NAFLD, and gut dysbiosis is considered a hit. Thus, controlling gut microbiota is a potential target in the management of NAFLD, and probiotics can be used as a treatment agent for NAFLD. The current study aimed to investigate the efficacy of probiotics against nonalcoholic steatohepatitis in a hepatocyte-specific PTEN knockout mouse model that mimics the characteristics of human NAFLD. Probiotics were administered to male knockout mice for 8 or 40 weeks. Next, we assessed hepatic inflammation, fibrosis, carcinogenesis, and oxidative stress. Probiotics were found to reduce serum transaminase levels, NAFLD activity score, and the gene expression of pro-inflammatory cytokines. In addition, they decreased liver fibrosis grade, which was examined via Sirius red staining, gene expression of fibrotic markers, and hydroxyproline. Furthermore, probiotics suppressed the number of liver tumors, particular in HCC. Probiotics reduced oxidative stresses, including glutathione levels, and anti-oxidative stress marker, which may be an underlying mechanism for their beneficial effects. In conclusion, probiotics treatment had beneficial effects against NAFLD and carcinogenesis in hepatocyte-specific PTEN knockout mice.

## Introduction

Nonalcoholic fatty liver disease (NAFLD), a hepatic feature of metabolic syndrome, is characterized by the accumulation of triglycerides in hepatocytes. Nonalcoholic steatohepatitis (NASH), an advanced form of NAFLD, is clinically important because it can progress to cirrhosis and hepatocellular carcinoma (HCC)^1^. Recently, the number of patients with NAFLD has been increasing in different countries worldwide including Japan. Therefore, NAFLD has become a major liver disease in clinical practice. However, effective therapeutic agents for NAFLD have not been developed.

The multiple-hit theory is a promising mechanism for NAFLD pathogenesis^2^. In this theory, multiple risk factors, including gut microbiota, insulin resistance, dyslipidemia, and oxidative stress, are simultaneously involved in the development of NAFLD. Among them, gut dysbiosis is considered a hit. That is, small intestinal bacterial overgrowth is frequently observed in patients with NAFLD^3^, and the number of bacterial components derived from the gut increases in the blood^4^. Based on our previous studies, gut bacterial components stimulate Toll-like receptors (TLRs) and induce liver inflammation, resulting in the progression of NASH and NASH-related carcinoma^5–7^. Therefore, gut microbiota is a promising therapeutic target for NAFLD.

Probiotics treatment is used to modify the component of gut microbiota. Probiotics reduce the expression of TLR ligands in the gut and oxidative stress^8,9^. Animal studies showed that probiotics had beneficial effects against NAFLD. However, most studies investigated the early phase of NAFLD, including liver steatosis and inflammation, but not liver fibrosis, which is a feature during the late phase^10^. In addition, most carcinogenesis studies used chemically induced or ectopic models, in which the tumor cells were transplanted into the skin of immune-deficient mice^11^. These animal models do not always mimic the natural history of NAFLD. Therefore, the efficacy of probiotics must be validated using models that mimic the natural history of NAFLD, including steatohepatitis and then fibrosis and subsequent hepatocarcinogenesis.

The hepatocyte-specific PTEN knockout (PTEN KO) mice have been reported to show steatohepatitis, liver fibrosis, and subsequent carcinogenesis^12–14^. Moreover, it shows that the PTEN protein is downregulated in approximately 40% of HCCs in humans^15^. Thus, PTEN KO mice are suitable models for investigating the efficacy of probiotics. The current study showed that probiotics are effective against NASH and NASH-related carcinogenesis in PTEN KO mice.

## Results

### Probiotics reduced liver damage in PTEN KO mice

Table 1 shows the body weight and weight of each organ, after 8 and 40 weeks of probiotics treatment, respectively. In both 8- and 40 weeks probiotics treatment, PTEN KO mice had a significantly larger liver weight, but not body weight, than control mice. Probiotics did not affect the body or liver weight of PTEN KO mice in both 8- and 40 weeks probiotics treatment.

**Table 1:** Metabolic parameters in mice.

Mice	F/F		KO	
Treatment (8 weeks)	Vehicle	Probiotics	Vehicle	Probiotics
Number	n = 5	n = 5	n = 5	n = 5
BW (g. end point)	31.8 ± 1.0	30.8 ± 1.1	28.9 ± 0.4a	29.0 ± 0.9
Liver weight (g)	1.58 ± 0.08	1.30 ± 0.12	2.98 ± 0.11a	2.96 ± 0.26
Liver/BW (%)	4.96 ± 0.15	4.22 ± 0.27	10.3 ± 0.3a	10.19 ± 0.7
Epididymal fat (g)	0.48 ± 0.08	0.44 ± 0.05	0.4 ± 0.07	0.36 ± 0.05
Fat/BW (%)	1.51 ± 0.25	1.43 ± 0.16	1.38 ± 0.24	1.24 ± 0.21
Spleen weight (g)	0.11 ± 0.01	0.11 ± 0.01	0.19 ± 0.02a	0.17 ± 0.03
Spleen/BW (%)	0.35 ± 0.05	0.37 ± 0.03	0.67 ± 0.07a	0.60 ± 0.13

Mice	F/F		KO	
Treatment (40 weeks)	Vehicle	Probiotics	Vehicle	Probiotics
Number	n = 11	n = 7	n = 14	n = 16
BW (g. end point)	33.1 ± 7.3	30.4 ± 3.2	32.9 ± 2.7	31.3 ± 3.0
Liver weight (g)	1.71 ± 0.36	1.52 ± 0.17	4.91 ± 1.36a	4.23 ± 1.39
Liver/BW (%)	5.21 ± 0.84	5.03 ± 0.65	15.3 ± 3.8a	13.5 ± 4.1
Epididymal fat (g)	0.79 ± 0.85	0.46 ± 0.36	0.22 ± 0.11a	0.26 ± 0.14
Fat/BW (%)	2.06 ± 1.79	1.45 ± 0.92	0.68 ± 0.31a	0.80 ± 0.42
Spleen weight (g)	0.28 ± 0.22	0.19 ± 0.22	0.21 ± 0.10	0.19 ± 0.13
Spleen/BW (%)	0.92 ± 0.78	0.66 ± 0.83	0.69 ± 0.35	0.64 ± 0.56

Pten flox/flox mice (F/F) and PTEN KO mice (KO) were fed with normal water (vehicle) or probiotics for 8 or 40 weeks. BW, body weight; F/F, Pten flox/flox mice; KO, PTEN KO mice. Groups were analyzed using one-way analysis of variance and the Tukey post-hoc test. Data were presented as mean ± standard deviation. ^a^Significantly different from F/F among vehicles.

**Figure 1:**
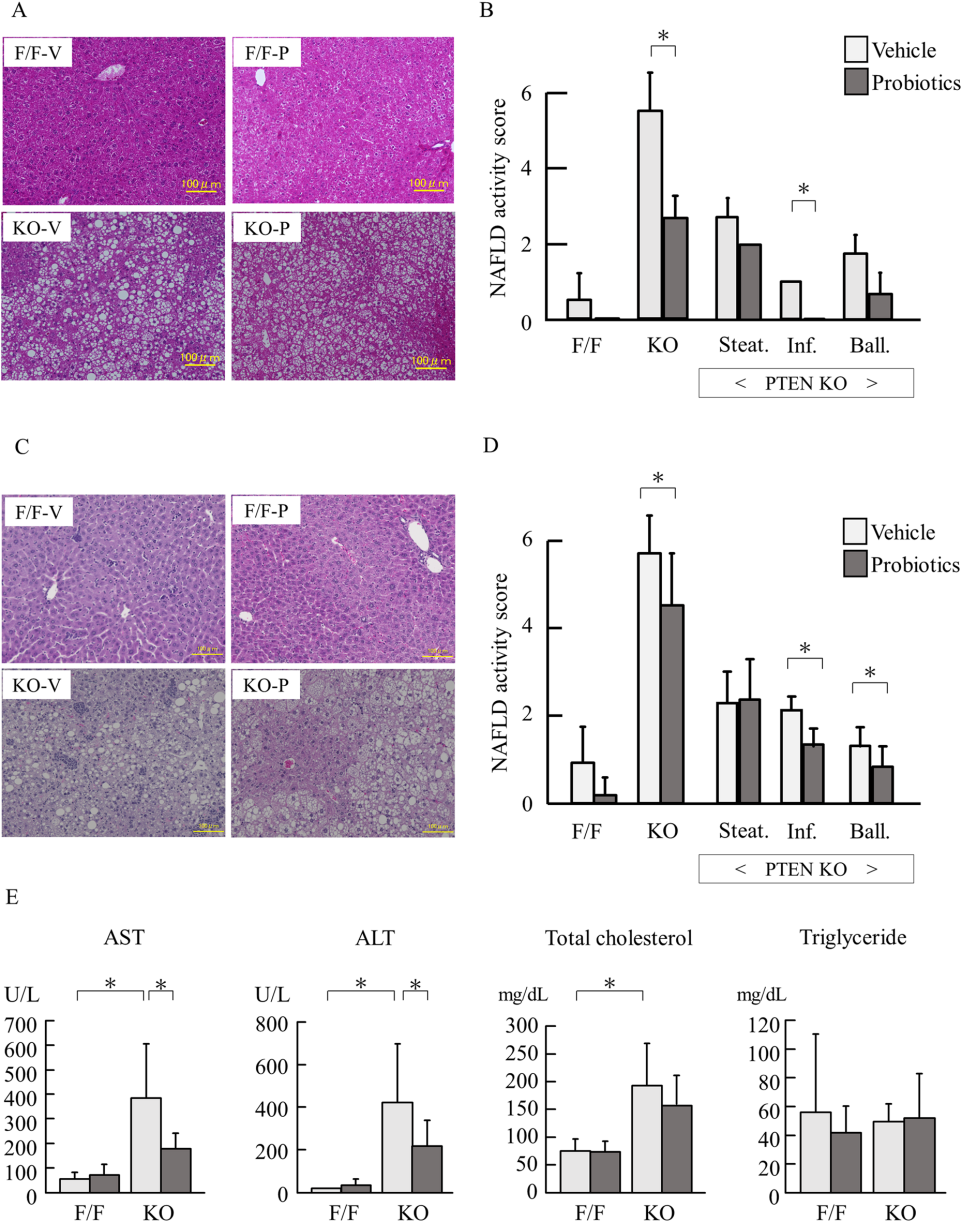
Histological and biological assessment (**A**) The representative of photos of HE staining of the liver in mice treated for 8 weeks probiotics. PTEN KO presented with steatosis, lobular inflammation, and hepatocyte ballooning. (**B**) NAFLD activity score (each group n = 5). In PTEN KO mice, scores of steatosis, inflammation (Inf.), and ballooning (Ball.) are shown. (**C**) The representative of photos of HE staining of the liver in mice treated for 40 weeks probiotics. PTEN KO presented with steatosis, lobular inflammation (Inf.), and hepatocyte ballooning (Ball). (**D**) NAFLD activity score (each group n = 7–16). In PTEN KO mice, scores of steatosis, inflammation, and ballooning are shown. (**E**) Serum levels of transaminases and lipids (each group n = 7–16, Experiments were performed in technically duplicate). Groups were analyzed using the student’s *t*-test. Data were presented as mean ± standard deviation. F/F, PTEN F/F mice; KO, PTEN KO mice; V, vehicle; P, probiotics; NAS, NAFLD activity score.*P < 0.05.

Then, we assessed histological findings using hematoxylin and eosin-stained specimens. Compared with control mice, PTEN KO mice presented with increased steatosis, lobular inflammation, and hepatocyte ballooning for 16 weeks of age (Fig. 1A). Indeed, the overall NAFLD activity score (NAS) was higher in PTEN KO mice than that in control mice, and probiotics treatment for 8 weeks reduced the overall score (Fig. 1B). Among three components of NAS, including steatosis, inflammation, and ballooning, probiotics treatment significantly reduced the score of lobular inflammation but not steatosis in PTEN KO mice (Fig. 1B). PTEN KO mice treated with 40 weeks of probiotics showed reduced score of inflammation and ballooning (Fig. 1C,D). Probiotics treatment significantly reduced the serum aspartate aminotransferase and alanine aminotransferase levels, which were elevated in PTEN KO mice (Fig. 1E). However, there was no significant difference in total cholesterol and triglyceride levels between the vehicle and probiotics treatment groups of PTEN KO mice.

### Probiotics reduced inflammatory responses and cell death in the liver

**Figure 2:**
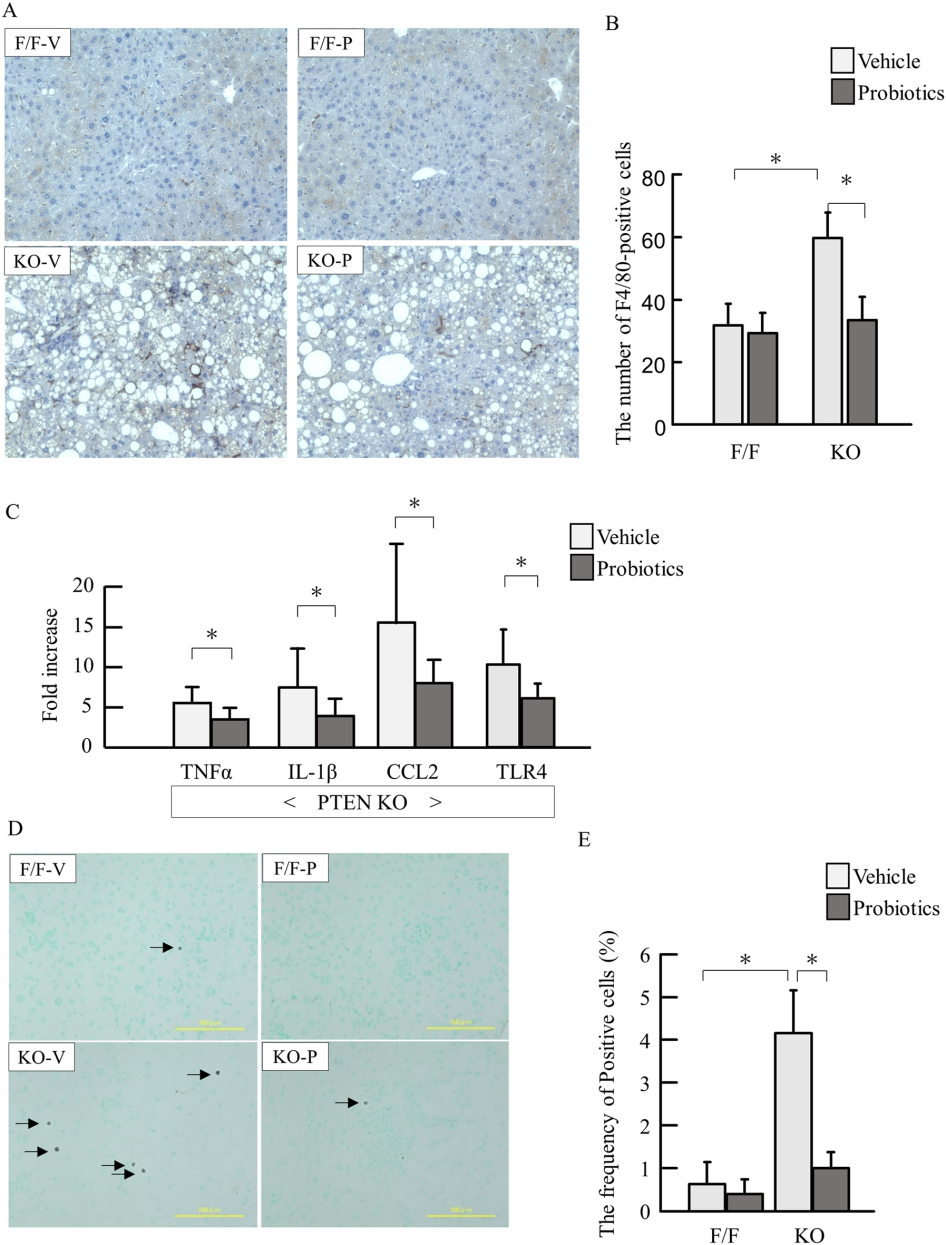
Hepatic inflammation and gene expression of cytokines. (**A**) Immunostaining for F4/80. F/F, PTEN F/F mice; KO, PTEN KO mice; V, vehicle; P, probiotics; (**B**) The number of F4/80-positive cells. (**C**) mRNA expression of inflammatory markers in PTEN KO mice (Experiments were performed in technically duplicate). (**D**) The representative photos of TUNEL staining. E. The frequency of TUNEL-positive cells. Groups were analyzed using the student’s *t*-test. Data were presented as mean ± standard deviation.*P < 0.05 (each group n = 9–11).

We then assessed inflammation in the liver. PTEN KO mice showed increased number of F4/80-positive cells in the liver. Probiotics treatment suppressed inflammatory cell infiltration examined by F4/80 staining (Fig. 2A,B). In according to histological examinations, probiotics treatment reduced mRNA expression of TNFα, IL-1β, CCL2, and TLR4 (Fig. 2C).

Then we examined the apoptotic cells by terminal deoxynucleotidyl transferase dUTP nick end labeling (TUNEL) staining. PTEN KO mice with vehicle showed increased frequency of TUNEL-positive hepatocytes, which was reduced by probiotics treatment (Fig. 2D,E).

### Probiotics reduced liver fibrosis

**Figure 3:**
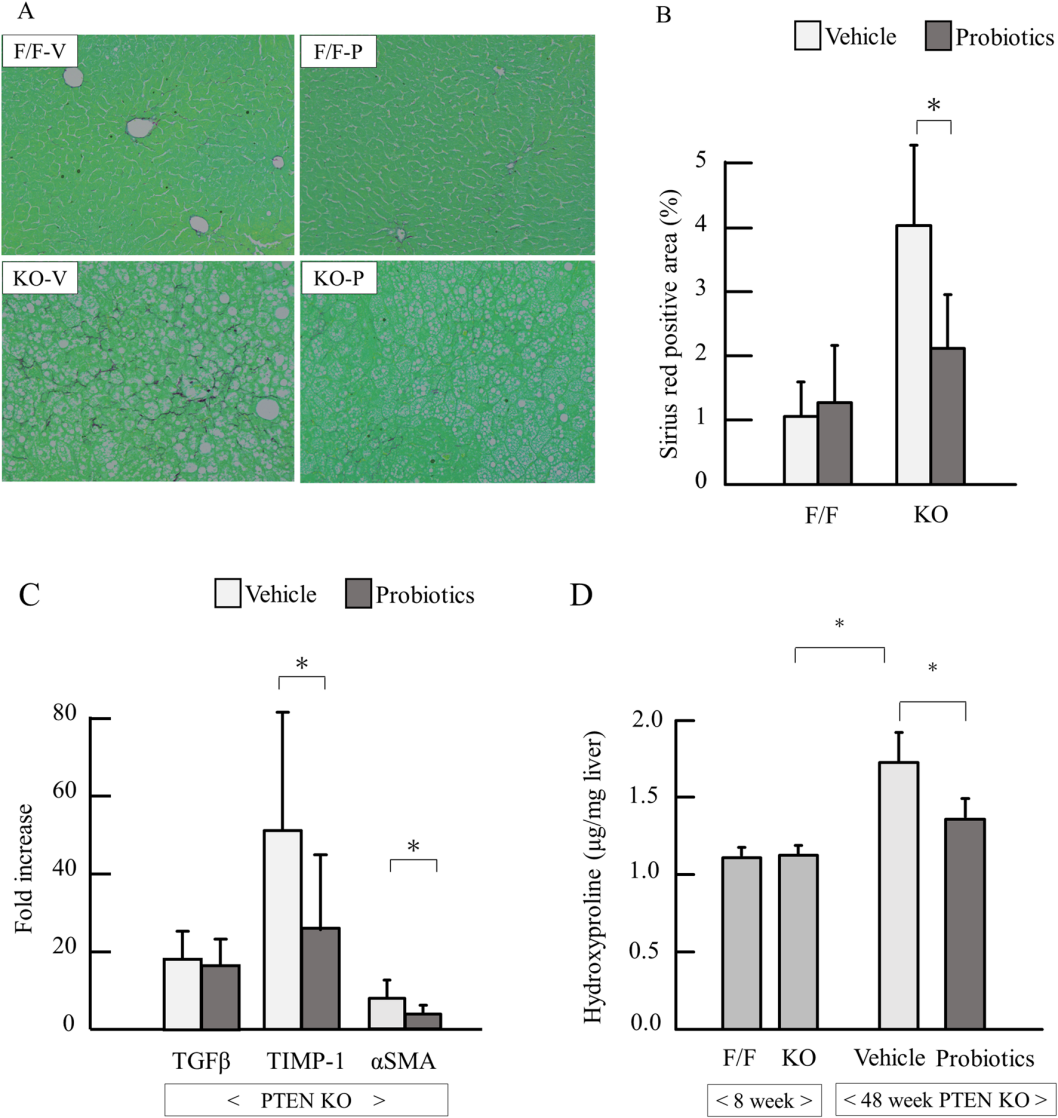
Assessment of liver fibrosis. (**A**) Sirius red staining of the liver. (**B**) Quantification of Sirius red-positive area (each group n = 5). (**C**) mRNA expression of fibrosis markers (each group n = 9–11). (**D**) The content of hepatic hydroxyproline. Groups were analyzed using the student’s *t*-test. Data were presented as mean ± standard deviation. *P < 0.05. (Experiments in **C** and **D** were performed in technically duplicate).

Then we assessed liver fibrosis in 40 weeks treatment of probiotics. In Sirius red staining, PTEN KO mice had increased liver fibrosis, which was attenuated by probiotics (Fig. 3A). Similar results were obtained by measuring the Sirius red-stained area (Fig. 3B). PTEN KO mice had a significantly higher mRNA expression of fibrotic markers, including TGFβ, TIMP-1, and alpha-SMA in the liver, and probiotics significantly reduced the expression of TIMP-1 and alpha-SMA (P < 0.05) but not TGFβ (Fig. 3C). In addition, probiotics treatment significantly reduced hydroxyproline content in the liver (Fig. 3D).

We further assessed the grade of steatohepatitis around tumors in PTEN KO mice at 48 weeks of age. The background of inflammation and fibrosis that close to tumors in vehicle group were more severe than in those in probiotics group (Supplemental Fig. 1).

### Probiotics suppressed oxidative stress

**Figure 4:**
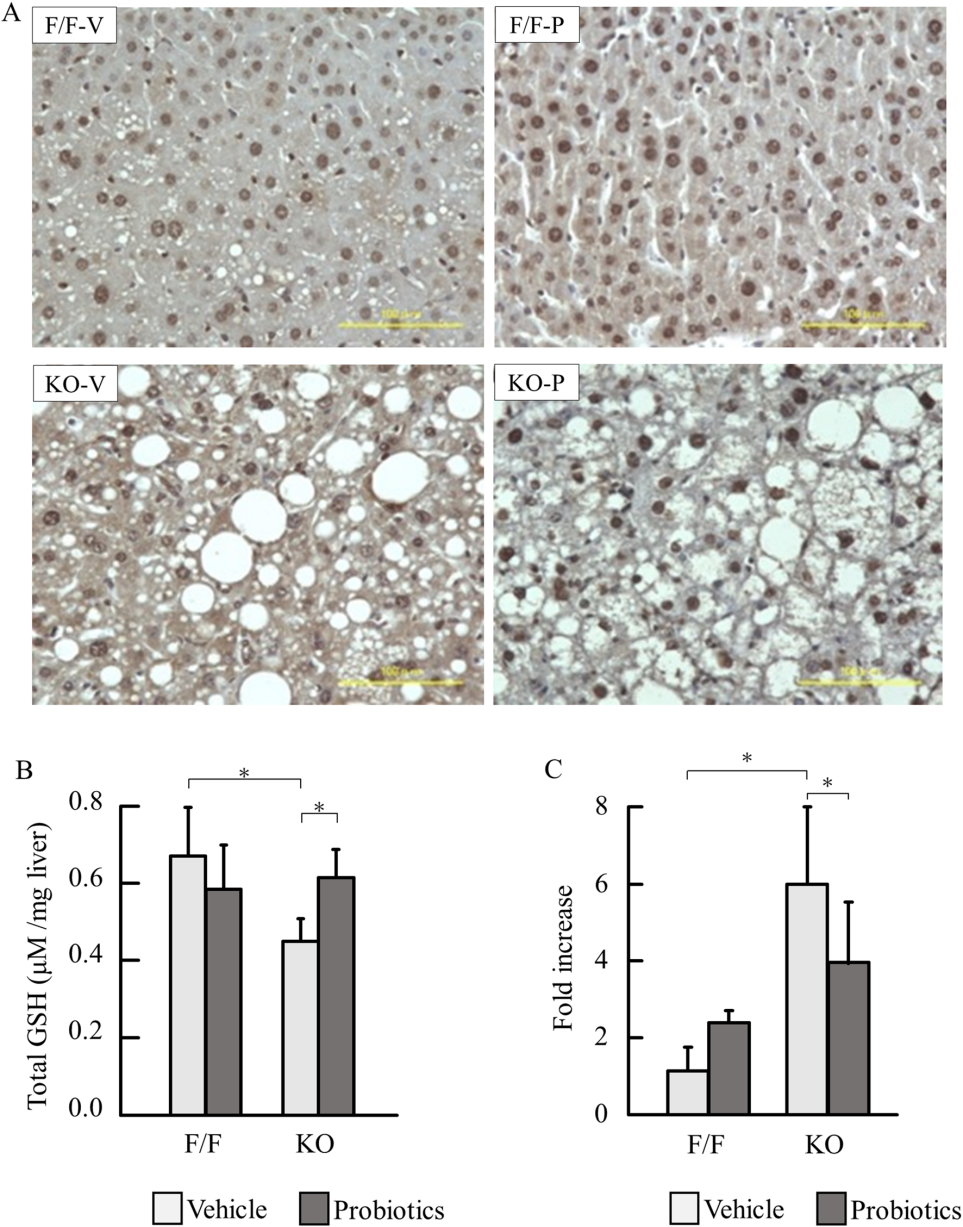
Assessment of oxidative stress in non-tumor tissue. (**A**) The representative photos of immunostaining for 8OHdG. (**B**) Total glutathione concentration (each group n = 9–11). (**C**) mRNA expression of GPx4 (each group n = 9–11). Groups were analyzed using one-way analysis of variance and the Tukey post-hoc test. Data were presented as mean ± standard deviation. GSH, glutathione-sulfhydryl. *P < 0.05 (Experiments in B and C were performed in technically duplicate).

We assessed oxidative stress markers. Cytoplasmic expression of 8OHdG has been reported as intense oxidative stress^16^. Hepatocytes in PTEN KO mice showed intense cytoplasmic expression of 8OHdG, which was reduced by probiotics treatment (Fig. 4A). Total glutathione concentration, a marker of antioxidant stress, was significantly reduced in PTEN KO mice compared with that of control mice (*P* < 0.001). Further, probiotics significantly restored glutathione concentrations (Fig. 4B) (P < 0.05). Then we examined oxidative-related genes. Among them, GPx4 was significantly increased in PTEN KO mice and probiotics treatment reduce it (Fig. 4C).

### Probiotics inhibited hepatocarcinogenesis

**Figure 5:**
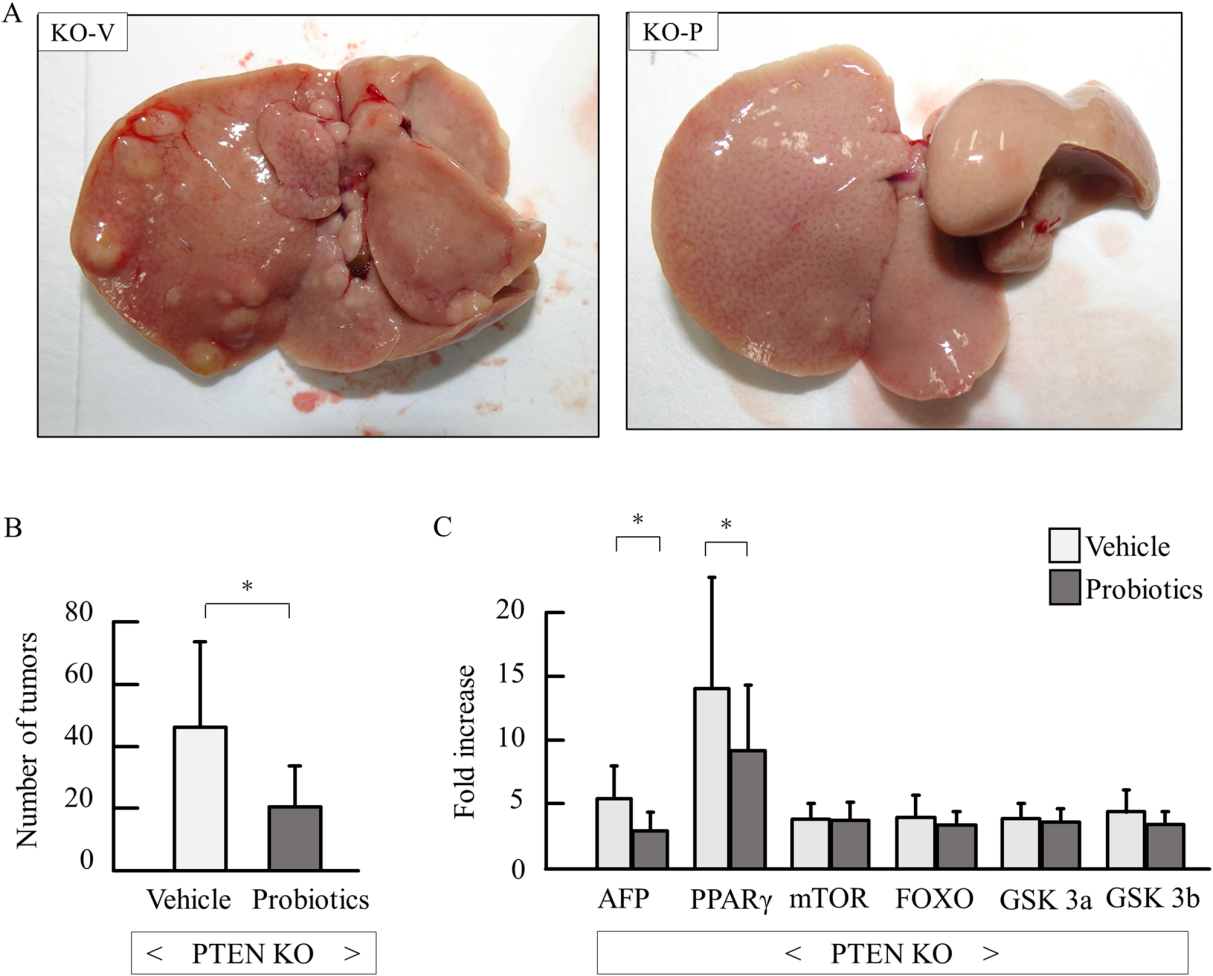
Macroscopic assessment of tumors and tumor-related genes. (**A**) Gross appearance of PTEN KO. (**B**) Number of tumors (each group n = 11–16). (**C**) mRNA expression of tumor- and pten-related genes (each group n = 9–11, Experiments were performed in technically duplicate). Groups were analyzed using the student’s *t*-test. Data were presented as mean ± standard deviation. *P < 0.05.

All PTEN KO mice with vehicle (14/14) and probiotics (16/16) developed liver tumors at 48 weeks of age. Although the maximum size of tumors was not different between vehicle and probiotics groups (data not shown), the tumor numbers of the liver surface were significantly reduced by probiotics treatment (Fig. 5A,B). Among tumor- and pten-related genes, alpha-fetoprotein and PPARγ genes were reduced by probiotics treatment (Fig. 5C).

**Figure 6:**
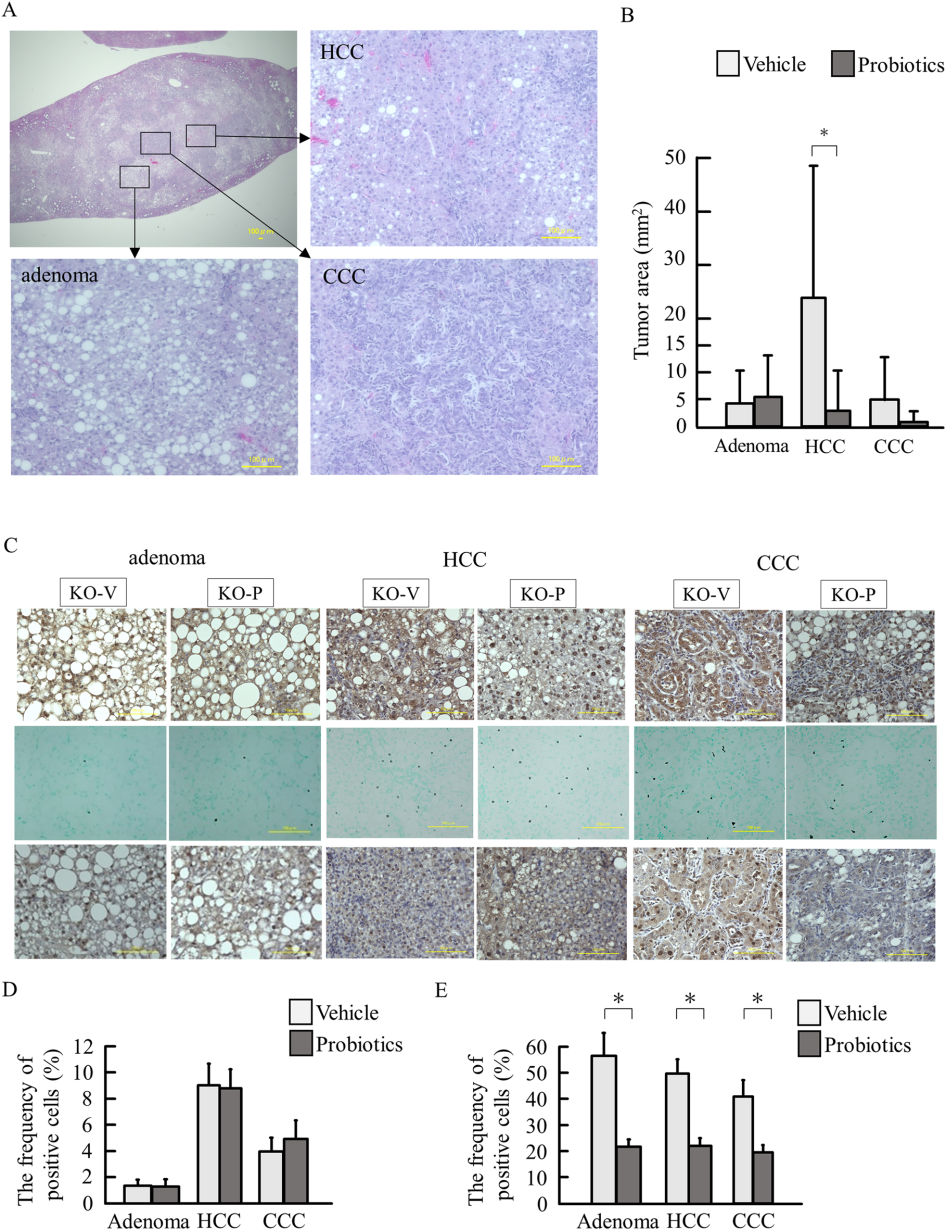
Histological assessment of tumors. (**A**) The representative HE staining of the liver tumor developed in PTEN KO mice. (**B**) The areas of each tumor, including adenoma, HCC, and CCC. (**C**) Staining for 8OHdG (upper), TUNEL (middle), and PCNA (lower). (**D**) The frequency of TUNEL-positive cells. (**E**) The frequency of PCNA-positive cells Groups were analyzed using one-way analysis of variance and the Tukey post-hoc test. Data were presented as mean ± standard deviation. *P < 0.05.

As we previously reported^17^, there were three types of tumors, including adenoma, HCC, and cholangiocellular carcinoma (CCC) (Fig. 6A). We measured the areas of these three types of tumors in HE specimens. Although probiotics did not suppress the area of adenomas, probiotics treatment significantly reduced the areas of HCCs (Fig. 6B). CCC tended to be reduced by probiotics treatment. Then we assessed the association between oxidative stress and cell behaviors in tumors. In PTEN KO mice with vehicle, intense staining of 8OHdG were noted in the cytoplasm of HCC and CCC, which were reduced by probiotics treatment (Fig. 6C). The numbers of tumor cells that underwent apoptosis were equivalent between vehicle and probiotics groups (Fig. 6C,D). In contrast, the frequency of proliferating cell nuclear antigen (PCNA)-positive cells increased in adenoma, HCC, and CCC, which was reduced probiotics treatment (Fig. 6C,E).

## Discussion

This study showed that probiotics treatment decreased liver damage, inflammation, fibrosis, and hepatocarcinogenesis in hepatocyte-specific PTEN KO mice. To the best of our knowledge, this research first showed that probiotics are effective based on an animal model that mimics the natural history of human NASH and hepatocarcinogenesis. Moreover, probiotics treatment reduced oxidative stress, an underlying mechanism of NAFLD.

Although several reports showed that probiotics are effective against NAFLD, the composition of probiotics varied. *Lactobacillus rhamnosus GG* and *L. plantarum NA136* were found to be effective based on the studies of Kim et al.^18^ and Zhao et al.^19^, respectively*.* Bubnov et al.^20^ revealed that the combination of several probiotic strains may have beneficial effects against NASH because each strain has its own strengths, such as suppression of weight gain and reduction of cholesterol level. In addition, probiotics can reduce the production of unfavorable substances including hydrogen sulfide by increasing the diversity of intestinal microflora. Furthermore, probiotics inhibited the transfer of unfavorable substances and gut bacteria into the bloodstream by protecting intestinal barrier^19^. The probiotics used comprised 20 different strains, accounting for multiple factors associated with the beneficial effects against NASH.

By contrast, the effect of probiotics varied among diet models even if the same probiotics were used. VSL#3 comprises eight bacteria including *L. casei*, *L. plantarum*, *L. acidophilus*, *L. delbrueckii subsp. bulgaricus*, *Bifidobacterium longum*, *B. breve*, *B. infantis*, *and Streptococcus salivarius* subsp.^21^ Velayudham et al.^22^ revealed that VSL#3 was effective against liver fibrosis but not fatty liver damage in mice fed with MCD diet. In contrast, it was efficient against fat deposition and liver damage in mice fed with HFD^23^. Our previous study showed that the intestinal microflora changed based on diet^24^. The current study used a normal diet, thereby indicating that diet had minimal contribution to NASH.

In the current study, probiotics had no inhibitory effects against dyslipidemia and liver steatosis in PTEN KO mice. Nonetheless, liver damage, inflammation, and fibrosis were significantly suppressed. In general, NASH results in inflammation and fibrosis after excessive accumulation of fat in the liver^25^. However, the amount of fat in the liver is not always correlated with inflammation and fibrosis. Yamaguchi et al.^26^ showed that the inhibition of diacylglycerol acyltransferase 2, an enzyme involved in triglyceride synthesis, improved liver steatosis but worsened inflammation and fibrosis in mice fed with MCD diet. Gluchowski et al.^27^ revealed that hepatocyte-specific diacylglycerol acyltransferase 2 KO mice improved fatty liver but not in insulin resistance, inflammation, and fibrosis. Quality rather than quantity of fat is another aspect of the development of NASH^17,28^. Free cholesterol in the liver aggravates liver damage^29^. Although free cholesterol in the liver was not assessed in the present study, it plays a potential role in NASH pathogenesis.

The current study reproduced our previous findings that inflammation and liver fibrosis promote the progression of hepatocarcinogenesis, particular in HCC^17^. In addition, these inflammation and fibrosis were more severe around tumors, suggesting that these factors facilitate the growth of tumors. Our study confirmed that probiotics inhibited hepatocarcinogenesis in PTEN KO mice by suppressing background inflammation and fibrosis. Probiotics facilitated histological improvement in the background liver in aflatoxin- or DEN-induced chemical carcinogenesis models, which also induce HCC^30,31^. In addition, Li et al.^32^ showed that probiotics suppressed angiogenesis by reducing IL-17 in a tumor-transplanted model. Thus, probiotics may contribute to tumor development indirectly and directly. However, these animal models were chemically induced models or an ectopic tumor-transplanted model. Although STAM mice, PPARα KO mice, DAIAMND mice, and MUP-uPA transgenic mice are used in orthotopic carcinogenic models^10^, little data is available on the effects of probiotics on these mice.

A mechanism in which probiotics inhibit inflammation, fibrosis, and carcinogenesis, thereby reducing oxidative stress, was considered. Indeed, oxidative stress is enhanced in several NASH models^9,33^. The hepatic hydroxy peroxidase concentrations of PTEN KO mice were sevenfold higher than those of wild-type^12^. Hence, persistent oxidative stress may contribute to the development of steatohepatitis and liver tumors. Interestingly, oxidate stress showed different functions in non-tumor tissue and tumor tissue. Namely, oxidative stress contributed to cell proliferation in tumors. In contrast, oxidative stress contributed to cell death in non-tumor tissue. In the current study, the content of glutathione, an antioxidant, decreased in the liver of PTEN KO mice. The glutathione levels in the plasma and liver reduced in human NAFLD^34^. Luz et al.^35^ revealed that higher ROS, NO, and lipopolysaccharide levels from enteric bacteria can deplete liver glutathione levels by decreasing the expression of S-adenosylmethionine and methionine adenosyltransferase, which are essential for glutathione synthesis. Although we measured serum LPS levels, it did not elevate enough to see differences.

Although the current study showed that probiotics suppressed NASH and NASH carcinogenesis, it had several limitations. Recent studies showed that some microRNA down regulate PTEN expression in patients with NAFLD^36^, suggesting that a subset of patients with NAFLD are associated with PTEN. PTEN KO mice mimic the natural history of human NASH but at lower risk for obesity^10^. In general, patients with NAFLD present with obesity. Further, non-obese NASH has a more severe course than obese NASH^37^. PTEN KO mice experienced severe inflammation, liver fibrosis, and carcinogenesis. Thus, probiotics may have favorable effects regardless of obesity. In the current study, we used commercially available probiotics. The type of strains and whether a combination of strains contribute to the effects of probiotics were not evaluated. Therefore, further studies should be conducted to determine the component and amount of probiotics.

In conclusion, probiotics treatment reduced oxidative stress and inhibited NASH and NASH carcinogenesis in hepatocyte-specific PTEN KO mice, which mimic the natural history of human NAFLD. Therefore, probiotics may be useful in clinical practice.

## Materials and methods

### Animals

Pten flox/flox (Pten F/F) mice and albumin-cre (Alb-Cre) recombinase transgenic mice were purchased from Jackson Laboratory (the USA). Pten f/f mice were crossed with C57Bl/6 mice for at least 10 generations. Hepatocyte-specific PTEN knockout mice were generated by crossing Alb-Cre recombinase transgenic mice. All mice had a C57Bl/6 background. Male mice were used in the current study. Alb-Cre recombinase-negative Pten f/f mice were used in the control group. All mice were provided with free access to food and water until the end of the experiments.

Mice were fed with a standard chow (MFG-LID; Oriental Yeast Co., Ltd., Tokyo, Japan) and housed with a 12:12 h light/dark cycle until 16 weeks or 48 weeks of age. Pten f/f mice (n = 5–11/group) and PTEN KO mice (n = 5–16/group) were randomly assigned to vehicle or probiotic groups. The probiotic groups were fed with 4 g/L of probiotics (THT Mix 20 strains; Sceti, Tokyo, Japan) suspended in drinking water for 8 weeks or 40 weeks (from 8 weeks of age), which was replaced every three days. The probiotics contained 20 species of lactic acid-producing bacteria at 1 billion cfu/g (Supplemental Table 1). Mice were killed at the age of 16 or 48 weeks, and harvested samples were stored at − 80 °C until use. Blood was taken from portal vein. The livers were divided into three lobes, and visible tumors with a diameter of > 1 mm were counted. The largest love was further divided into 5 pieces, then the middle part and the two edges were used in histological assessment of tumors. Animal experiments were approved by the Review Board of Jichi Medical University (20061-01; Tochigi, Japan). All animals received humane care according to the criteria outlined in the Guide for the Care and Use of Laboratory Animals prepared by the National Academy of Science and our institutions and carried out in compliance with the ARRIVE guidelines.

### Histology

Hematoxylin and eosin staining and Sirius red staining were performed according to the published protocols^5^. The NAS was evaluated according to the study of Kleiner et al.^38^ To assess Sirius red-positive areas, 10 slides at a magnification of (×200) per field of view were prepared for each sample and quantified using Image J (U.S. National Institutes of Health, Bethesda, Maryland, the USA)^39^.

Immunostaining for F4/80 (eBioscience, MA, the USA), PCNA (proteinech, IL, the USA), TUNEL (R&D, MN, the USA), and 8-OHdG (Nikken Seil, Tokyo, Japan) were performed as published protocol according to the manufacturer’s instructions. Histological assessment was blindly performed by two hepatologists under assistance of a pathologist.

### Measurement for transaminase and lipid levels

Serum aspartate aminotransferase, alanine aminotransferase, total cholesterol, and triglyceride levels were assessed using FUJI DRI-CHEM SLIDE (FUJIFILM, Kanagawa, Japan), according to the manufacturer’s instructions.

### Quantitative real-time polymerase chain reaction

RNA was extracted from the liver using TRI Reagent (Sigma-Aldrich, St. Louis, MO, the USA). The extracted RNA was converted to cDNA via reverse transcription. The cDNA was then subjected to PCR using the primers listed (Supplemental Table 2) and TB GREEN premix Ex Taq (Takara, Tokyo, Japan). Gene expression was normalized to that of 18S RNA as an internal control.

### Measurement of total glutathione-sulfhydryl concentration and hydroxyproline

In total, 100 mg of mouse liver was collected in 1 mL of 5% 5-sulfosalicylic acid (FUJIFILM, Osaka, Japan) and then homogenized and centrifuged to collect the supernatant. The supernatant was diluted 10 times with distilled water, and the sample was evaluated. The total glutathione-sulfhydryl concentration was assessed using the GSSG/GSH Quantification Kit (Dojindo Molecular Technologies Inc., Kumamoto, Japan), according to the manufacturer’s instructions. Absorbance was evaluated using Multiskan FC (Thermo Scientific, Massachusetts, the USA) with a 405-nm filter, and the concentration was calculated using the Pseudo-endpoint method.

For measurement of hydroxyproline, a total of 10 mg was used and processed according to the manufacturer’s instructions (Sigma-Aldrich).

### Statistical analyses

Statistical analyses were performed using the *t*-test or Mann–Whitney *U*-test, one-way ANOVA or Kruskal–Wallis test, and post-hoc test (the Tukey or Steel–Dwass method). All statistical analyses were performed with EZR (Saitama Medical Center, Jichi Medical University, Saitama, Japan)^40^, which is a graphical user interface for R (The R Foundation for Statistical Computing, Vienna, Austria). More precisely, it is a modified version of R commander designed to add statistical functions frequently used in biostatistics. A P value of < 0.05 was considered statistically significant.

## Supplementary Information

Supplementary Information.

## Data Availability

The data are presented with in paper. Additional raw data are available on request from the corresponding author.
