# Supplementary material for: Probiotics suppress nonalcoholic steatohepatitis and carcinogenesis progression in hepatocyte-specific PTEN knockout mice
Source: Sci Rep. 2022 Sep 28;12:16206. doi: 10.1038/s41598-022-20296-3 (PMC9519992; doi:10.1038/s41598-022-20296-3)
Supplement: Supplementary file 1 — Supplementary Information. [file 41598_2022_20296_MOESM1_ESM.docx]

Supplementary information

Supplementary Table 1: Recipe of probiotics

|  | species |  |  | species |
| --- | --- | --- | --- | --- |
| 1 | *Bifidobacterium bifidum* |  | 11 | *Lactobacillus delbr bulgaricus* |
| 2 | *Bifidobacterium infantis* |  | 12 | *Lactobacillus casei* |
| 3 | *Bifidobacterium animalis lactis* |  | 13 | *Oenococcus oeni* |
| 4 | *Bifidobacterium longum* |  | 14 | *Lactococcus lactis* |
| 5 | *Lactbacillus fermentum* |  | 15 | *Leuconostoc mesenteroides* |
| 6 | *Lactobacillus plantarum* |  | 16 | *Streptococcus thermophilus* |
| 7 | *Lactobacillus rhamnosus* |  | 17 | *Pediococcus pentosaceus* |
| 8 | *Lactobacillus paracasei* |  | 18 | *Staphylococcus carnosus* |
| 9 | *Lactobacillus acidophilus* |  | 19 | *Staphylococcus xylosus* |
| 10 | *Lactobacillus brevis* |  | 20 | *Enterococcus faecium* |

Supplementary Table 2: Sequence of primers for quantitative real-time PCR

| Primers | Forward | Reverse |
| --- | --- | --- |
| 18s | AGTCCCTGCCCTTTGTACACA | CGATCCGAGGGCCTCACTA |
| TNFα | AGGGTCTGGGCCATAGAACT | CCACCACGCTCTTCTGTCTAC |
| IL-1β | GGTCAAAGGTTTGGAAGCAG | TGTGAAATGCCACCTTTTGA |
| CCL2 | ATTGGGATCATCTTGCTGGT | CCTGCTGTTCACAGTTGCC |
| TIMP-1 | AGGTGGTCTCGTTGATTTCT | GTAAGGCCTGTAGCTGTGCC |
| TGFβ | GTGGAAATCAACGGGATCAG | ACTTCCAACCCAGGTCCTTC |
| GPx4 | GCCTGGATAAGTACAGGGGTT | CATGCAGATCGACTAGCTGAG |
| TLR4 | TGTCATCAGGGACTTTGCTG | TGTTCTTCTCCTGCCTGACA |
| αSMA | GTTCAGTGGTGCCTCTGTCA | ACTGGGACGACATGGAAAAG |
| AFP | CAGCAGCCTGAGAGTCCATA | GGCGATGGGTGTTTAGAAAG |
| PPARγ | TCTTCCATCACGGAGAGGTC | GATGCACTGCCTATGAGCAC |
| mTOR | CAGTTCGCCAGTGGACTGAAG | GCTGGTCATAGAAGCGAGTAGAC |
| FOXO 1 | GGGTCCCACAGCAACGATG | CACCAGGGAATGCACGTCC |
| GSK 3a | ATTATGCGTAAGCTGGACCAC | CGTCTCGGGCACATACTCC |
| GSK 3b | TGGCAGCAAGGTAACCACAG | CGGTTCTTAAATCGCTTGTCCTG |


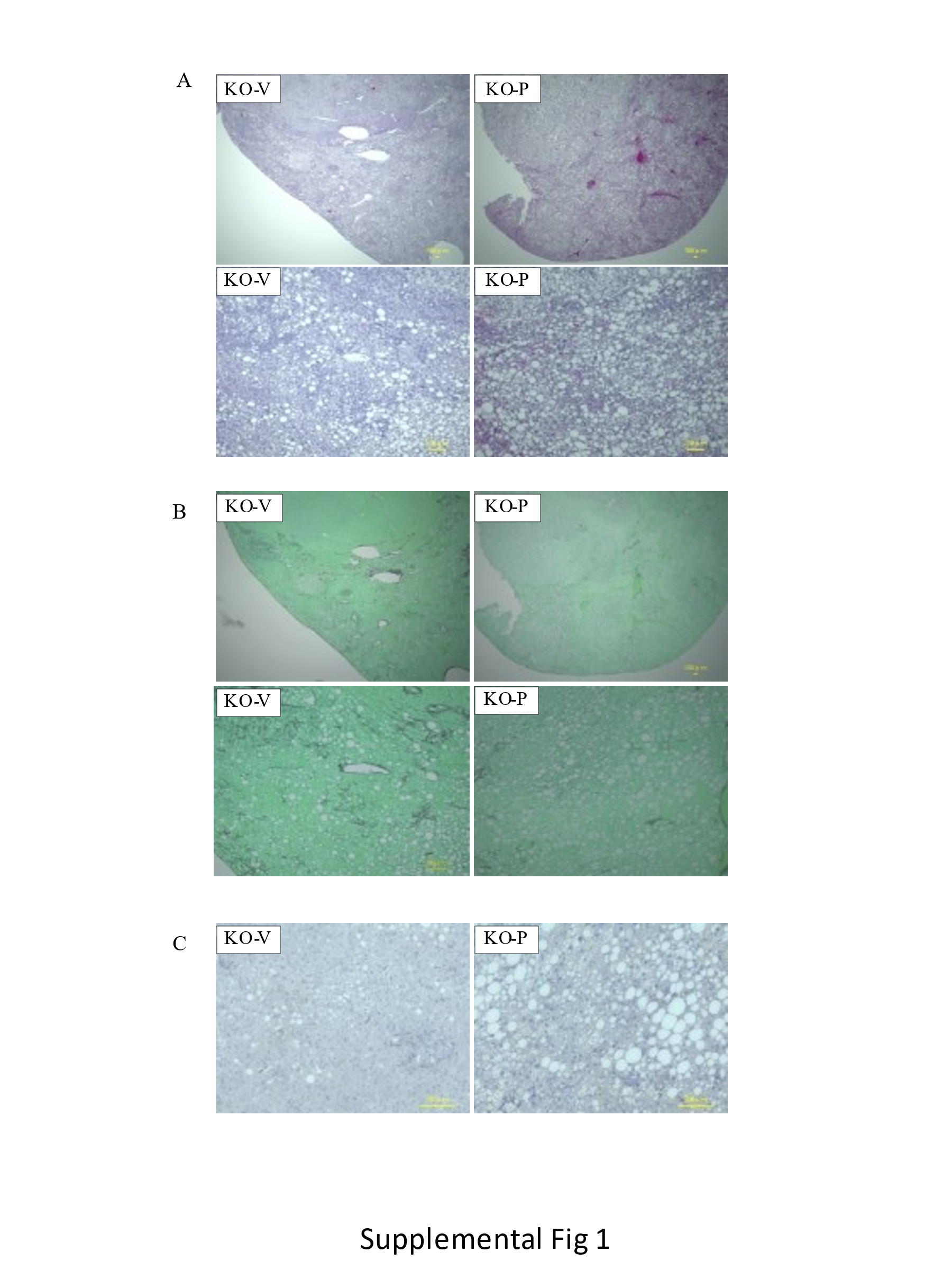


**Supplemental Figure 1. Histological assessment of non-tumor area that close to tumors.** A: The representative photos of HE staining (upper, low-power view; lower, high-power view). B: The representative photos of Sirius Red staining (upper, low-power view; lower, high-power view). C. The representative photos of F4/80 staining. F/F, PTEN F/F mice, KO, PTEN KO mice; V, vehicle; P, probiotics.
